# Growth performance, blood variables, intestinal bacterial content, and morphological measurements of broilers supplemented with *Lactobacillus casei*-fermented mixture of red rice and aromatic ginger

**DOI:** 10.14202/vetworld.2022.818-826

**Published:** 2022-04-02

**Authors:** Fitri Dwi Astuti, Sugiharto Sugiharto, Turrini Yudiarti, Endang Widiastuti, Hanny I. Wahyuni, Tugay Ayaşan

**Affiliations:** 1 Department of Animal Science, Faculty of Animal and Agricultural Sciences, Universitas Diponegoro, Semarang, Central Java, Indonesia; 2 Osmaniye Korkut Ata University, Kadirli Faculty of Applied Sciences, Department of Organic Farming Business Management, Osmaniye, Turkey

**Keywords:** broiler, fermentation, health, phytogenic, prebiotic, probiotic

## Abstract

**Background and Aim:**

Natural feed additives are important for broiler sustainability during the post-antibiotic era. This study aimed to evaluate the possible effects of the dietary supplementation of *Lactobacillus casei*-fermented mixture of red rice and aromatic ginger (FERMIX) on growth, blood profiles, intestinal bacterial content, and morphological measurements of broilers.

**Materials and Methods:**

Two hundred broiler chicks were allotted to four groups with five replications, including CONTROL (chicks provided with basal feed), FERMIX025 (basal feed supplemented with 0.25% FERMIX), FERMIX050 (0.50% FERMIX), and FERMIX100 (1.00% FERMIX). FERMIX is an anaerobic fermentation product from a mixture of red rice flour and aromatic ginger using *L. casei*. Blood, organs, digesta, and carcass were collected on day 35.

**Results:**

Final body weight, weight gain, feed intake, and feed conversion ratio did not differ (p>0.05) across treatments. FERMIX at 0.5% increased (p<0.05) spleen relative weight. Liver weight was lower (p<0.05) in broilers fed with 1% FERMIX. The liver weight linearly reduced (p<0.05) with the increased FERMIX levels. FERMIX at 0.25% elevated (p<0.05) broiler drumsticks’ yield than that in other groups. Erythrocytes, hemoglobin, packed cell volume, and plasma total protein levels were quadratically higher (p<0.05) in FERMIX050 treated than in other groups. Increased FERMIX levels resulted in a linear decrease (p=0.08) in ileal lactose-negative *Enterobacteriaceae* counts. Villi height/crypt depth (CD) ratio was quadratically higher (p<0.05) in the duodenum of broiler supplemented with 0.5% FERMIX. The jejunal CD was quadratically lower (p<0.05) in FERMIX050 than in other groups.

**Conclusion:**

Although it did not influence growth, dietary FERMIX, especially at 0.5%, improved immune competencies, physiological conditions, and health of broilers’ intestine.

## Introduction

Broiler chicken is an essential sector of the animal protein supply chain and a significant contributor to the Indonesian economy. However, with the prohibition on antibiotic growth promoter (AGP) usage in feed, the broiler industry has begun facing sustainability issues. Continuous AGP usage can lead to the establishment of antibiotic resistance and detrimentally impact consumer health [1]. Literature suggests that adding feed additives to broiler feed can help them grow faster and stay healthier. The use of probiotics as a feed additive reduces the number of pathogenic bacteria in broiler intestines and improves feed digestibility, resulting in a favorable effect on the growth rate [2,3]. Prebiotics are another feed additives that can help probiotics function better in boosting the population of non-pathogenic bacteria in broiler chicken intestines [1]. In addition to probiotics and prebiotics, studies have documented the potential of herbal plants as feed additives for broiler chickens due to their contents of antioxidants, antimicrobials, and immunostimulants [4].

*Lactobacillus casei* is a Gram-positive bacteria widely employed as a probiotic for broiler chickens [5]. These bacteria can maintain the microbial balance, improving the digestive and absorptive functions of broiler intestines. In addition, *L. casei* can modulate the immune response of broilers [5]. In addition to being a probiotic, *L. casei* is extensively used as a fermentation starter in the feed industry [6]. In some countries, including Indonesia, red rice (*Oryza sativa* L.) is a popular cereal, with carbohydrates serving as key nutrients [7,8]. In addition to anthocyanins, red rice contains non-starch polysaccharides and resistant starch, which may act as prebiotics to help probiotic microbes proliferate [8,9]. In the case of resistant starch, Ashwar *et al*. [10] reported that resistant starch derived from rice starch is an effective delivery carrier for oral probiotic administration. In addition to being a prebiotic, red rice might be used to deliver *L. casei* to broiler chickens. Aromatic ginger (*Kaempferia galanga* L.) is a *Kaempheria* species widely used in traditional medicine worldwide [11]. The rhizome of *K. galanga* contains several pharmacologically active compounds, including phenylpropanoids, flavanols, and terpenoids [12]. With antimicrobial activity [12,13], antioxidants [14], and anti-inflammatory activities [14], *K. galanga* rhizome may be exploited as a phytogenic feed additive for broilers. According to a recent study, fermentation enhances the bioavailability of bioactive components (such as polyphenols) and the synthesis of health-promoting products [15].

At present, the use of *L. casei*-fermented blends of red rice and aromatic ginger (FERMIX) in broiler chickens has never been revealed. This study aimed to evaluate the effect of dietary supplementation of *L. casei*-fermented mixture of red rice and aromatic ginger on growth performance, blood profiles, intestinal bacterial content, and morphological measurements of broilers. It was hypothesized that feeding FERMIX could improve broilers’ growth performance, physiological conditions, and intestinal health.

## Materials and Methods

### Ethical approval

The *in vivo* study was approved by the Animal Ethics Committee of the Faculty of Animal and Agricultural Sciences, Universitas Diponegoro (No. 57-08/A3/KEP/FPP).

### Study period and location

The *in vivo* study was conducted during April and May 2021 at the Faculty of Animal and Agricultural Sciences, Universitas Diponegoro, Semarang, Central Java, Indonesia.

### Preparation of dietary supplements

FERMIX production was modified from Mangisah *et al*. [16]. Briefly, the pure *L. casei* isolate was rejuvenated on Man, Rogosa, and Sharpe (MRS) agar and anaerobically incubated for 2 days at 38°C. Skim milk (20 g) was diluted in 180-mL distilled water and swirled until homogenous as a growth medium for bacteria (source of lactose). The *L. casei* isolates (two plates) were harvested with skim milk (200 mL) and incubated at 38°C for 2 days in anaerobic conditions. To produce FERMIX, 50-g brown rice flour and 50-g aromatic ginger were mixed and placed in an anaerobic jar, dissolved in 200-mL skim milk containing *L. casei* (as prepared previously), and swirled until homogenous. The mixture was then incubated at 38°C for 2 days under anaerobic conditions. The mixture was then sun-dried after fermentation, obtained for proximate and microbiological analysis. The proximate analysis was conducted according to the standard method described by Association of Official Analytical Chemists [17]. The rest of the product was stored at 4°C until use for *in vivo* experiment. The proximate analysis showed that the supplement contained (on a dry matter basis) 11.4% moisture, 11.5% crude protein, 1.86% crude fat, 1.96% crude fiber, and 4.45% ash. Following the anaerobic incubation on MRS agar for 2 days at 38°C, the supplement contained lactic acid bacteria (LAB) of 6.82×10^13^ cfu/g.

### *In vivo* experiments

Two hundred Lohmann broiler chicks were communally grown in brooding cages with commercial feed starting when they arrived at the broiler house. On day 8, the chicks (unsexed; body weight of 169.76±1.74 g; means±standard deviation) were divided into four groups of dietary treatments with five replicates/pens, each with ten chicks. The dietary groups included CONTROL (broiler chicks provided with basal feed), FERMIX025 (broiler chicks provided with basal feed supplemented with 0.25% FERMIX), FERMIX050 (broiler chicks provided with basal feed supplemented with 0.50% FERMIX), and FERMIX100 (broiler chicks provided with basal feed supplemented with 1.00% FERMIX). The FERMIX was added (“on top”) to basal feeds at the end of the mixing process. The mixing of feeds was conducted manually, and to achieve a homogeneous mixture, approximately 20-kg feed ingredients were mixed for every batch. The basal feeds were formulated (Table-1) [18] in mash form as starter and finisher feed when the chicks were 8-21 and 22-35 days old, respectively. The feed was formulated to comply with the nutritional requirement of chicks during the starter [19] and finisher [20] periods. From arrival to day 7, the birds were offered commercial pre-starter feed containing 23.0% crude protein, 5.0% crude fat, 5.0% crude fiber, and 7.0% ash (according to feed label). Feed and drinking water was offered *ad libitum* throughout the study period. Chickens were housed in an open-sided broiler house with plastic curtains, light bulbs, and fans to manually control the temperature and humidity inside the broiler house. The temperature was maintained at 28-30°C and humidity at 80-85%. Lighting was provided to the chickens throughout the day (24 h) during the study.

**Table 1:** Ingredients and chemical constituents of feed (days 8-35).

Items (%, unless otherwise noted)	Starter (days 8-21)	Finisher (days 22-35)
Yellow maize	53.4	60.9
Palm oil	2.35	3.05
Soybean meal (crude protein of 44.15%)	40.2	32.0
DL-methionine, 990 g	0.19	0.19
Bentonite	0.75	0.75
Limestone	1.00	1.00
Monocalcium phosphate	1.30	1.30
Premi×^1^	0.34	0.34
Chlorine chloride	0.07	0.07
Salt	0.40	0.40
Calculated chemical constituents		
ME (kcal/kg)^2^	2901	3024
Crude protein	22.0	19.0
Crude fiber	5.46	5.53
Ca	1.14	1.12
P (available)	0.57	0.58
Analyzed chemical constituents		
Moisture	14.4	14.6
Crude protein	19.1	14.4
Crude fat	3.59	3.75
Crude fiber	2.73	2.67
Ash	4.70	4.99

1 Provided per kg of feed=1100 mg Zn, 1000 mg Mn, 75 mg Cu, 850 mg Fe, 4 mg Se, 19 mg I, 6 mg Co, 1225 mg K, 1225 mg Mg, 1250,000 IU vit A, 250,000 IU vit D_3_, 1350 g pantothenic acid, 1875 g vit E, 250 g vit K_3_, 250 g vit B_1_, 750 g vit B_2_, 500 g vit B_6_, 2500 mg vit B_12_, 5000 g niacin, 125 g folic acid and 2500 mg biotin

2 ME (metabolizable energy) was calculated according to formula [18]=40.81 {0.87 [crude protein+2.25 crude fat+nitrogen-free extract]+2.5}

Recording of feed consumption and weighing of body weight were conducted weekly. Blood samples were collected from veins of the chicken wings (two chicks per pen/10 chicks for each treatment group) on day 35 and placed in a vacutainer tube containing ethylenediaminetetraacetic acid. After blood collection, chickens (one chick per pen) were slaughtered, feathered, and dissected. Then, the internal organs were removed and weighed empty. To count the number of bacteria in the intestine, researchers obtained samples from the ileum and cecum. Each 2 cm section of the duodenum, jejunum, and ileum was removed and placed in a tube filled with 10% buffered formalin to assess intestinal morphology. Total carcass and carcass proportion were also determined in this study. Briefly, the carcass was weighed after the feathers, head, feet, and giblet were removed. Following that, the carcass proportions (breast, wings, thigh, drumstick, and back) were determined. The total carcass was calculated using the following equation: (carcass weight [g]/live bird weight [g])×100. The carcass proportion was calculated using the following formula: (cut weight [g]/carcass weight [g])×100.

As previously described by Isroli *et al*. [21], the dilution flask method was employed to determine full blood counts in this study. A burker chamber was used to count corpuscles and compute the numbers of erythrocytes and leukocytes. Hematocrit levels were calculated using the microhematocrit method. Differential leukocytes were counted using a light microscope with an immersion lens. The coverslip procedure was utilized during the preparation of blood smears. The measurement of total protein in the plasma was conducted as described by Bianchi-Bosio [22] using the biuret method. In principle, the cupric ion reacts with protein in an alkaline environment to form a purple complex. The absorbance of this complex is proportional to the sample’s protein concentration.

Sugiharto *et al*. [23] described the bacterial population in the intestine was measured. The amount of coliform and lactose negative *Enterobacteriaceae* was assessed as red and colorless colonies on MacConkey agar (Merck KGaA, Darmstadt, Germany) after a 24 h aerobic incubation at 38°C. Coliform and lactose-negative *Enterobacteriaceae* were summed to determine *Enterobacteriaceae*. The amount of LAB on De MRS agar (Merck KGaA) was estimated after anaerobic incubation at 38°C for 48 h. The histological measurement of the small intestinal segment was conducted as outlined by Tunç *et al*. [24]. Staining of 5-m intestinal slices was performed using hematoxylin and eosin. The villi heights (VH) and crypt depths (CD) were measured using an optical microscope connected to a digital camera. The average VH and CD were calculated using five measurements for each bird.

### Statistical analysis

One-way analysis of variance (one-way ANOVA, Statistical Package for the Social Sciences [SPSS] 16.0 version; SPSS Inc., Chicago, IL, USA) was used to statistically assess the experiment’s data, which was based on a completely randomized design. The Duncan multi-range test was used after a significant effect of treatments was detected. In addition, the influence of additives at different levels in feeds was determined using an orthogonal polynomial contrast test for linear and quadratic effects. Before the ANOVA test, the homogeneity tests were conducted to confirm that the populations had the same distribution (normal distribution). A significant effect was considered when p<0.05. When 0.05≤p<0.10 was observed, the tendency was considered.

## Results and Discussion

### Growth performance of broilers

Improvement in growth has been observed in broilers with dietary administration of probiotics, prebiotics, and herbal products [1,16]. In contrast to the previous study, data obtained in this investigation exhibited no FERMIX effect at various levels in diets on broiler chicken productivity indicators (p>0.05). The regression test further showed no linear impact (p>0.05) of treatments on the growth performance of broilers (Table-2). In line with the present results, Fajardo *et al*. [6] found no effect of probiotic *L. casei* on broiler chickens’ growth, feed consumption, and feed conversion ratio. Likewise, Rehman *et al*. [25] showed no influence of multistrain probiotics (mixture of *Lactobacillus plantarum*, *Enterococcus faecium*, *Lactobacillus rhamnosus*, *Candida pintolepesii*, *Aspergillus oryzae*, and *Bifidobacterium bifidum*) on growth, feed consumption, and feed efficiency of broilers during day 0-35 of the experiment. Concerning prebiotics, dietary administration of mannan-oligosaccharides showed no effect on the weight and feed efficiency of broilers during the starter (day 0-21) and finisher (day 22-35) periods in the investigation of Rehman *et al*. [25]. In addition, Sarangi *et al*. [26] reported no influences of dietary inclusion of prebiotic (mannan-oligosaccharides), probiotic (*L. plantarum*, *Lactobacillus bulgaricus*, *Bifidobacterium bifidus*, *Streptococcus faecium*, and *Saccharomyces cerevisiae*), and synbiotic (mixture of *L. bulgaricus*, *S. faecium*, *L. plantarum*, *S. cerevisiae*, *B. bifidus*, and mannan-oligosaccharides) on final weight, weight gain, feed consumption, and feed conversion of broilers during the rearing for 6 weeks. Disparities in results between studies are likely due to differences in probiotic species and strains, prebiotic types and sources, doses of probiotics and prebiotics included in feeds, and experimental conditions. [16,26]. In the case of *K. galanga*, research on the utilization of this herbal plant as a broiler growth promoter remains lacking. Indeed, in terms of the efficacy of dietary herbal supplementation on growth performance, the growth-promoting effect of herbal products may be highly dependent on the types and natures of herbs used and the amounts given to chicks [27,28].

**Table 2:** Growth performance of broiler chickens.

Items	CONTROL	FERMIX025	FERMIX050	FERMIX100	SEM	p-value		

						A	L	Q
Final BW, g/bird	1501	1505	1604	1512	24.9	0.44	0.57	0.35
BW gain, g/bird	1329	1338	1435	1342	24.8	0.42	0.46	0.32
Feed intake, g/bird	2699	2807	2791	2763	35.1	0.74	0.60	0.37
FCR	2.03	2.10	1.95	2.09	0.04	0.61	0.96	0.69

BW=Body weight, FCR=Feed conversion ratio, CONTROL=Broiler chicks provided with basal feed, FERMIX025=Basal feed supplemented with 0.25% FERMIX, FERMIX050=Basal feed supplemented with 0.50% FERMIX, FERMIX100=Basal feed supplemented with 1.00% FERMIX, A=Analysis of variance, L=Orthogonal polynomial contrasts test for linear effects, Q=Orthogonal polynomial contrasts test for quadratic effects, SEM=Standard error of the means

### Internal organs of broilers

The current investigation showed that dietary FERMIX, particularly at 0.5% of feed, enhanced (p<0.05) chicken spleen weight (Table-3). The increased spleen weight (relative to live body weight) is mostly associated with the improved immune response of broilers. In an earlier study, Sikandar *et al*. [29] documented an increase in spleen relative weight with the inclusion of probiotic *Bacillus subtilis*, an indicator of the improved immune responses in disease-free broilers. Consistent with this, Salam *et al*. [30] documented broiler’s increased spleen relative weight with supplementation of black cumin powder. In addition to the *Bursa of fabricius* and thymus, the spleen is the vital immune organ responsible for the orientation and maturation of the immune cell. In healthy animals, the increased immune organ weight is attributed to enhanced immune cell proliferation, which indicates better immune competencies [31]. According to Sikandar *et al*. [29], the higher spleen weight may result from amplified B and T lymphocytes. Here, the capability of the probiotic microorganism in modulating the immune response of broilers could be the reason for the increased spleen relative weight in broilers. In line with this, the potential of prebiotics and herbal products in modulating the immune responses and intestinal bacterial populations [1] contribute to the higher relative weight of the spleen in broilers. Data in the current work showed that relative liver weight was lower (p<0.05) in broilers fed with 1% FERMIX in diet than that in other birds (Table-3). The rationale behind this condition was unknown, but it seemed that the elevated levels of fiber in diets with the increasing FERMIX were responsible for the lower liver weight. These inferences were supported by the orthogonal polynomial contrast test for linear effect, showing that the liver weight decreased (p<0.05) with the increased levels of FERMIX in broiler diets. Previously, Mohiti-Asli *et al*. [32] documented that dietary fiber reduced liver weight, mainly due to the decreased fatty acid synthesis and thus accumulation of lipid in the liver. The orthogonal polynomial contrast test for quadratic effects revealed that broilers fed 0.25% FERMIX had a tendency (p=0.07) to have a higher relative heart weight. Although the exact cause of this condition remains undetermined, the higher metabolic rate in broilers due to FERMIX treatment may increase the heart muscle activity and consequently heart weight. However, this inference should be treated with caution because the higher heart relative weight was not found in FERMIX050 and FERMIX100 birds.

**Table 3:** Internal organ relative weights of broiler chickens.

Items (% live BW)	CONTROL	FERMIX025	FERMIX050	FERMIX100	SEM	p-value		

						A	L	Q
Heart	0.36	0.43	0.37	0.36	0.01	0.10	0.44	0.07
Liver	2.28^a^	2.17^a^	2.33^a^	1.66^b^	0.09	0.02	0.02	0.08
Proventriculus	0.50	0.44	0.52	0.45	0.02	0.53	0.69	0.85
Gizzard	1.39	1.55	1.37	1.52	0.05	0.39	0.60	0.96
Pancreas	0.25	0.32	0.21	0.23	0.02	0.32	0.38	0.56
Duodenum	0.55	0.55	0.65	0.52	0.03	0.53	0.97	0.32
Jejunum	1.81	1.33	1.30	1.39	0.10	0.20	0.13	0.13
Ileum	0.93	1.00	0.91	0.93	0.05	0.91	0.79	0.82
Caeca	0.65	0.66	0.46	0.62	0.04	0.16	0.34	0.26
Abdominal fat	0.92	1.59	0.95	0.86	0.19	0.50	0.63	0.33
Spleen	0.14^b^	0.13^b^	0.30^a^	0.16^b^	0.02	0.04	0.22	0.15
Thymus	0.16	0.23	0.15	0.15	0.02	0.31	0.57	0.35
*Bursa of fabricius*	0.12	0.11	0.09	0.11	0.01	0.83	0.58	0.56

^a,b^ Means in the same row with different superscripts differ significantly (p<0.05). CONTROL=Broiler chicks provided with basal feed, FERMIX025=Basal feed supplemented with 0.25% FERMIX, FERMIX050=Basal feed supplemented with 0.50% FERMIX, FERMIX100=Basal feed supplemented with 1.00% FERMIX, A=Analysis of variance; L=Orthogonal polynomial contrasts test for linear effects, Q=Orthogonal polynomial contrasts test for quadratic effects, SEM=Standard error of the means

### Carcass traits of broilers

In this study, it was discovered that including FERMIX in the feed, especially at 0.25%, enhanced (p<0.05) the proportion of broiler drumsticks compared to the other treatments (Table-4). This finding indicated that at 0.25% of diet, FERMIX was able to increase the protein synthesis in broilers. Data regarding the plasma total protein confirmed this inference, at which dietary FERMIX increased the concentration of the total protein plasma of broilers (Table-5). It was most likely that bioactive components in FERMIX, including probiotics, prebiotics, and phytogenic, caused improved protein digestibility and utilization [16], resulting in increased protein synthesis and muscle deposition. However, this inference should be taken with care as other carcass traits (eviscerated carcass, breast, thigh, wings, and back) of broiler did not differ (p>0.05) among the groups.

**Table 4:** Carcass traits of broiler chickens.

Items	CONTROL	FERMIX025	FERMIX050	FERMIX100	SEM	p-value		

						A	L	Q
Eviscerated carcass (% live BW)	65.9	62.4	67.0	68.5	1.06	0.20	0.18	0.24

**% eviscerated carcass**								

Breast	35.7	35.5	34.2	34.8	0.68	0.88	0.55	0.79
Wings	11.7	13.5	11.8	11.0	0.49	0.30	0.36	0.18
Thigh	16.2	17.1	15.8	14.8	0.56	0.57	0.31	0.42
Drumstick	14.8^b^	17.2^a^	14.6^b^	15.0^b^	0.38	0.03	0.39	0.12
Back	21.6	16.7	23.5	24.5	1.49	0.25	0.23	0.33

^a,b^ Means in the same row with different superscripts differ significantly (p<0.05). BW=Body weight, CONTROL=Broiler chicks provided with basal feed, FERMIX025=Basal feed supplemented with 0.25% FERMIX, FERMIX050=Basal feed supplemented with 0.50% FERMIX, FERMIX100=Basal feed supplemented with 1.00% FERMIX, A=Analysis of variance; L=Orthogonal polynomial contrasts test for linear effects, Q=Orthogonal polynomial contrasts test for quadratic effects, SEM=Standard error of the means

**Table 5:** Blood parameters of broiler chickens.

Items	CONTROL	FERMIX025	FERMIX050	FERMIX100	SEM	p-value		

						A	L	Q
Erythrocytes (10^6^/mL)	2.36^b^	2.75^a^	2.78^a^	2.64^a^	0.05	<0.01	0.04	0.03
Leukocytes (10^3^/mL)	9.44	10.5	11.1	10.8	0.39	0.46	0.17	0.42
Hemoglobin (g/dL)	7.40	7.80	7.74	7.52	0.07	0.18	0.62	0.04
Packed cell volume (%)	21.5^b^	25.0^a^	25.5^a^	23.8^ab^	0.54	0.03	0.10	0.01
Heterophils (%)	34.5	31.9	31.9	31.9	1.74	0.94	0.62	0.72
Lymphocytes (%)	57.9	59.7	60.1	61.2	1.70	0.93	0.51	0.93
Monocytes (%)	7.40	8.00	7.70	6.90	0.39	0.80	0.62	0.39
H/L ratio	0.66	0.58	0.59	0.55	0.05	0.91	0.52	0.88
Plasma total protein (g/dL)	2.76^b^	3.31^a^	3.40^a^	3.18^a^	0.08	0.01	0.03	0.01

^a,b^ Means in the same row with different superscripts differ significantly (p<0.05). H/L ratio=Heterophils to lymphocytes ratio, CONTRL=Broiler chicks provided with basal feed, FERMIX025=Basal feed supplemented with 0.25% FERMIX, FERMIX050=Basal feed supplemented with 0.50% FERMIX, FERMIX100=Basal feed supplemented with 1.00% FERMIX, A=Analysis of variance; L=Orthogonal polynomial contrasts test for linear effects, Q=Orthogonal polynomial contrasts test for quadratic effects, SEM=Standard error of the means

### Blood parameters of broilers

It was clear in this investigation that erythrocyte levels were higher (p<0.05) in FERMIX treated compared to that in control. Indeed, the erythrocyte levels also linearly and quadratically increased (p<0.05) with the elevated FERMIX levels in diets. In line with erythrocytes, the values of hemoglobin and packed cell volume were also quadratically higher (p<0.05) in broilers administered 0.25 and 0.5% FERMIX in diets (Table-5). Given the key role of erythrocytes containing hemoglobin in transporting and delivering oxygen for cellular activity, greater erythrocyte values may be considered to enhance the rate of energy production in the cells, resulting in a positive effect on broiler growth performance [33]. Although statistically insignificant, the fact that broilers fed with 0.5% FERMIX gained more weight than those in the control group supported this inference. Probiotic microorganisms enhance the values of erythrocytes and packed cell volume of broilers in Shah *et al*. [34]. Likewise, dietary administration of mannan oligosaccharide increased the levels of erythrocytes and packed cell volume in the blood of turkey, as reported by Cetin *et al*. [35]. In addition, a recent study highlighted that the mixture of garlic and *Lactobacillus acidophilus* could increase the levels of erythrocytes and packed cell volume of broilers in the study by Sunu *et al*. [36]. The mechanism by which FERMIX increased the values of erythrocytes and packed cell volume of broilers remains unclear. However, it was most likely that the content of probiotic, prebiotic, and phytogenic components in FERMIX improved protein digestibility and utilization, increasing the availability of protein used for erythrocyte synthesis. The active compounds in FERMIX, particularly herb (*K. galanga*), may also deal with the stress condition, probably causing erythrocyte cell lysis during the rearing [33]. Altogether, the protective and erythropoiesis-promoting effect of FERMIX were responsible for the higher erythrocytes and packed cell volume of broiler in this study. Plasma total protein concentration was greater (p<0.05) in FERMIX treated than in the control broiler in this trial. Indeed, the increased graded levels of FERMIX were accompanied by the linear and quadratic increased (p<0.05) values of plasma total protein. In most situations, the increased plasma total protein values may represent the increased protein synthesis in the liver, which is used for broiler growth [37]. As mentioned above, the improved protein digestibility and utilization due to FERMIX administration seemed to be attributed to the increased liver protein synthesis producing an increased concentration of blood total protein.

### Intestinal bacterial counts and morphology of broilers

Data on the numbers of coliform, lactose-negative *Enterobacteriaceae*, *Enterobacteriaceae*, LAB, and LAB to coliform ratio in the ileal and cecal digesta are presented in Table-6. In general, dietary interventions had no influence (p>0.05) on these above selected bacterial counts, both in the ileum and cecum. However, increased amounts of FERMIX in broiler diets caused a linear decrease (p=0.08) in the number of lactose-negative *Enterobacteriaceae* in the ileum. Considering the potential pathogenicity of lactose-negative *Enterobacteriaceae*, the decrease in such a bacterial population may be beneficial for broilers’ health condition. The antibacterial properties of probiotics, prebiotics, and herbal products [1,5,12] in FERMIX were most likely to decrease the lactose-negative *Enterobacteriaceae* proliferation in the ileum of broiler in this investigation.

**Table 6:** Selected intestinal bacterial population of broiler chickens.

Items	CONTROL	FERMIX025	FERMIX050	FERMIX100	SEM	p-value		

						A	L	Q
Ileum								
Coliform	7.20	7.58	5.86	7.69	0.34	0.22	0.93	0.29
LNE	8.24	7.26	6.16	6.16	0.45	0.32	0.08	0.59
*Enterobacteriaceae*	8.22	7.94	6.16	7.95	0.42	0.30	0.49	0.22
LAB	10.6	10.4	10.3	11.4	0.19	0.20	0.18	0.11
LAB/coliform ratio	1.52	1.41	1.83	1.53	0.08	0.27	0.52	0.53
Cecum								
Coliform	7.91	7.57	7.55	6.76	0.24	0.39	0.12	0.64
LNE	8.35	6.83	7.88	6.90	0.39	0.46	0.36	0.74
*Enterobacteriaceae*	8.76	8.13	7.99	7.64	0.29	0.62	0.21	0.82
LAB	10.9	10.8	10.5	9.91	0.28	0.63	0.24	0.61
LAB/coliform ratio	1.37	1.45	1.45	1.51	0.06	0.91	0.50	0.96

cfu=Colony-forming unit, LNE=Lactose-negative *Enterobacteriaceae*, LAB=Lactic acid bacteria, CONTROL=Broiler chicks provided with basal feed, FERMIX025=Basal feed supplemented with 0.25% FERMIX, FERMIX050=Basal feed supplemented with 0.50% FERMIX, FERMIX100=Basal feed supplemented with 1.00% FERMIX, A=Analysis of variance; L=Orthogonal polynomial contrasts test for linear effects, Q=Orthogonal polynomial contrasts test for quadratic effects, SEM=Standard error of the means

Observation on the duodenum, jejunum, and ileum segments revealed no substantial influence (p>0.05) of feeding FERMIX on the intestinal morphology of broilers. The increased levels of FERMIX in diets did not linearly alter VH, CD, and VH to CD ratio (VH/CD) of the intestine (Table-7). However, there was a trend (p=0.09) that VH/CD was higher in the duodenum of broilers supplemented with 0.5% FERMIX. In addition, the orthogonal polynomial contrast test revealed that the VH/CD in FERMIX050 was quadratically higher (p<0.05) than in other birds. A higher VH/CD of the duodenum is attributed to the enhanced nutrient absorption capacity of broilers. In this study, the higher duodenal VH/CD of broilers supplemented with 0.5% agreed with the higher body weight of the respective chickens, although the values were not statistically significant. It seemed that probiotic, prebiotic, and phytogenic activities were responsible for the improved VH/CD in the broiler duodenum, as previously documented by Awad *et al*. [38] and Mohiti-Asli *et al*. [39]. In the jejunum, the CD was quadratically lower (p=0.05) in FERMIX050 than in other groups. According to Chacher *et al*. [40], decreasing the CD could signify efficient tissue turnover and good gut health. Indeed, the balance between pathogenic and beneficial bacteria increases the length of the villi and decreases the depth of the crypt [40]. Hence, it could be assumed that the probiotic, prebiotic, and phytobiotic activities of FERMIX, especially at 0.5% of diets, could balance the pathogenic and beneficial bacteria, resulting in better gut health.

**Table 7:** Intestinal morphology of broiler chickens.

Items	CONTROL	FERMIX025	FERMIX050	FERMIX100	SEM	p-value		

						A	L	Q
Duodenum								
Villi height (mm)	1087	1325	1150	1055	58.5	0.39	0.61	0.17
Crypt depth (mm)	125	117	96.2	122	5.71	0.29	0.57	0.14
VH/CD	8.73	11.9	12.3	8.68	0.69	0.09	0.96	0.01
Jejunum								
Villi height (mm)	1032	876.2	1015	969.8	56.5	0.80	0.93	0.65
Crypt depth (mm)	115	92.8	91.2	113	5.40	0.27	0.87	0.05
VH/CD	9.32	9.60	11.6	8.61	0.69	0.51	0.98	0.27
Ileum								
Villi height (mm)	622	840	509	598	61.1	0.28	0.46	0.59
Crypt depth (mm)	89.4	101	82.2	94.7	4.66	0.56	0.94	0.97
VH/CD	6.97	8.41	6.05	6.40	0.50	0.39	0.38	0.59

VH=Villi height, CD=Crypt depth, CONTRL=Broiler chicks provided with basal feed, FERMIX025=Basal feed supplemented with 0.25% FERMIX, FERMIX050=Basal feed supplemented with 0.50% FERMIX, FERMIX100=Basal feed supplemented with 1.00% FERMIX, A=Analysis of variance; L=Orthogonal polynomial contrasts test for linear effects, Q=Orthogonal polynomial contrasts test for quadratic effects, SEM=Standard error of the means

## Conclusion

Although it did not influence growth, dietary FERMIX, notably at 0.5%, improved broiler immune competencies, as seen by higher spleen relative weight. The physiological conditions were also improved by FERMIX at 0.5% of diets, as seen by higher levels of erythrocytes, hemoglobin, packed cell volume, and plasma total protein. The dietary treatment also improved the health of the intestine by lowering the counts of lactose-negative *Enterobacteriaceae*, raising the duodenal VH/CD, and decreasing the depth of the jejunal crypt.

## Authors’ Contributions

SS: Outlined the experiment, analyzed data, and drafted the manuscript. TY, EW, and HIW: Conducted broiler trial. TA: Designed the experiment and revised the manuscript. All authors read and approved the final manuscript.

## References

[1] Sugiharto S (2016). Role of nutraceuticals in gut health and growth performance of poultry. J. Saudi Soc. Agric. Sci.

[2] Pertiwi H, Mahendra M.Y.N (2021). Probiotic *Lactobacillus* sp. improved performance of broiler. Syst. Rev. Pharm..

[3] Sapsuha Y, Suprijatna E, Kismiati S, Sugiharto S (2021). Combination of probiotic and phythobiotic as an alternative for antibiotic growth promoter for broilers chickens a review. Livest. Res. Rural Dev.

[4] Sugiharto S (2021). Herbal supplements for sustainable broiler production during post-antibiotic era in Indonesia an overview. Livest. Res. Rural Dev.

[5] Zhang L, Zhang R, Jia H, Zhu Z, Li H, Ma Y (2021). Supplementation of probiotics in water beneficial growth performance, carcass traits, immune function, and antioxidant capacity in broiler chickens. Open Life Sci.

[6] Fajardo P, Pastrana L, Méndez J, Rodríguez I, Fuciños C, Guerra N.P (2012). Effects of feeding of two potentially probiotic preparations from lactic acid bacteria on the performance and faecal microflora of broiler chickens. Sci. World J.

[7] Priya T.S.R, Nelson A.R.L, Ravichandran K, Antony U (2019). Nutritional and functional properties of coloured rice varieties of South India:A review. J. Ethnic Food.

[8] Silva A.D.A, Ferreira M.V.D, Lima R.H.P, Barbosa J.L, Moreira L.B.M, Barbosa M.I.M (2020). Chemical characterization of the antioxidant, antihyperglycemic and antihypertensive capacities of red rice (*Oryza sativa* L.) whole flour. Rev. Chil. Nutr.

[9] Tantipaiboonwong P, Pintha K, Chaiwangyen W, Chewonarin T, Pangjit K, Chumphukam O, Kangwan N, Suttajit M (2017). Anti-hyperglycaemic and anti-hyperlipidaemic effects of black and red rice in streptozotocin-induced diabetic rats. ScienceAsia.

[10] Ashwar B.A, Gani A, Gani A, Shah A, Masoodi F.A (2018). Production of RS4 from rice starch and its utilization as an encapsulating agent for targeted delivery of probiotics. Food Chem.

[11] Srivastava N, Singh S, Gupta A.C, Shanker K, Bawankule D.U, Luqman S (2019). Aromatic ginger (*Kaempferia galanga* L.) extracts with ameliorative and protective potential as a functional food, beyond its flavor and nutritional benefits. Toxicol. Rep.

[12] Mans D.R.A, Djotaroeno M, Friperson P, Pawirodihardjo J (2019). Phytochemical and pharmacological support for the traditional uses of *Zingiberacea* species in Suriname a review of the literature. Pharmacogn. J.

[13] Tewtrakul S, Yuenyongsawad S, Kummee S, Atsawajaruwan L (2005). Chemical components and biological activities of volatile oil of *Kaempferia galanga* Linn. Songklanakarin J. Sci. Technol..

[14] Elshamy A.I, Mohamed T.A, Essa A.F, Abd-ElGawad A.M, Alqahtani A.S, Shahat A.A, Yoneyama T, Farrag A.R.H, Noji M, El-Seedi H.R, Umeyama A, Paré P.W, Hegazy M.E.F (2019). Recent advances in *Kaempferia* phytochemistry and biological activity:A comprehensive review. Nutrients.

[15] Annunziata G, Arnone A, Ciampaglia R, Tenore G.C, Novellino E (2020). Fermentation of foods and beverages as a tool for increasing availability of bioactive compounds. Focus on short-chain fatty acids. Foods.

[16] Mangisah I, Yunianto V.D, Sumarsih S, Sugiharto S (2021). Supplementation of garlic powder and *Lactobacillus casei* to improve nutrient digestibility, physiological conditions, and performance of broiler during starter phase. J. Indones. Trop. Anim. Agric..

[17] Association of Official Analytical Chemists (1995). Official Methods of Analysis.

[18] Bolton W (1967). Poultry Nutrition. MAFF Bulletin No.174.

[19] SNI (Indonesian National Standard) (2015). Standard for Broiler Feed:Starter Period (SNI 8173.2:2015).

[20] SNI (Indonesian National Standard) (2015). Standard for Broiler Feed:Finisher Period (SNI 8173.3:2015).

[21] Isroli I, Yudiarti T, Widiastuti E, Sugiharto S (2017). Probiotic *Bacillus* plus vitamins and minerals enhanced haemoglobin values and relative weight of ileum and improved feed conversion ratio of broilers during brooding period. Livest. Res. Rural Dev.

[22] Bianchi-Bosisio A, Worsfold P, Townshend A, Poole E (2005). Proteins:Physiological samples. Encyclopedia of Analytical Science.

[23] Sugiharto S, Yudiarti T, Isroli I, Widiastuti E, Wahyuni H.I, Sartono T.A (2018). The effect of fungi origin probiotic *Chrysonilia crassa* in comparison to selected commercially used feed additives on broiler chicken performance, intestinal microbiology, and blood indices. J. Adv. Vet. Anim. Res.

[24] Tunc M.A, Yildirim S, Yörük M.A (2019). Effects of tarragon (*Artemisia dracunculus*) powder on broiler performance parameters and histopathology of internal organs. Austral. J. Vet. Sci.

[25] Rehman A, Arif M, Sajjad N, Al-Ghadi M.Q, Alagawany M, Abd El-Hack M.E, Alhimaidi A.R, Elnesr S.S, Almutairi B.O, Amran R.A, Hussein E.O.S, Swelum A.A (2020). Dietary effect of probiotics and prebiotics on broiler performance, carcass, and immunity. Poult Sci.

[26] Sarangi N.R, Babu L.K, Kumar A, Pradhan C.R, Pati P.K, Mishra J.P (2016). Effect of dietary supplementation of prebiotic, probiotic, and synbiotic on growth performance and carcass characteristics of broiler chickens. Vet. World.

[27] Abdel-Azeem A.S, Basyony M.M (2019). Some blood biochemical, antioxidant biomarkers, lipid peroxidation, productive performance and carcass traits of broiler chicks supplemented with *Alpinia galangal* rhizomes extract during heat stress. Egypt. Poult. Sci.

[28] Kiramang K, Hidayat M.N, Anas A, Thaha A.H, Hafsan, Mappanganro R (2019). Effectivity of liquid herbal and supplemented frequency on the body weight percentage of the carcass and abdominal fat of broilers. IOP Conf. Ser. Earth Environ. Sci..

[29] Sikandar A, Zaneb H, Younus M, Masood S, Aslam A, Shah M, Rehman H (2017). Growth performance, immune status and organ morphometry in broilers fed *Bacillus subtilis*-supplemented diet. S. Afr. J. Anim. Sci..

[30] Salam S, Sunarti D, Isroli I (2013). Physiological responses of blood and immune organs of broiler chicken fed dietary black cumin powder (*Nigella sativa*) during dry seasons. J. Indones. Trop. Anim. Agric..

[31] Teo A.Y, Tan H.M (2007). Evaluation of the performance and intestinal gut microflora of broilers fed on corn-soy diets supplemented with *Bacillus subtilis* PB6 (CloSTAT). J. Appl. Poult. Res..

[32] Mohiti-Asli M, Shivazad M, Zaghari M, Aminzadeh S, Rezaian M, Mateos G.G (2012). Dietary fibers and crude protein content alleviate hepatic fat deposition and obesity in broiler breeder hens. Poult Sci.

[33] Sugiharto S (2020). Alleviation of heat stress in broiler chicken using turmeric (*Curcuma longa*) a short review. J. Anim. Behav. Biometeorol.

[34] Shah S.M.T, Islam M.T, Zabin R, Roy P.C, Meghla N.S, Jahid I.K (2021). Assessment of novel probiotic strains on growth, hematobiochemical parameters and production costs of commercial broilers in Bangladesh. Vet. World.

[35] Cetin N, Güçlü B.K, Cetin E (2005). The effects of probiotic and mannanoligosaccharide on some haematological and immunological parameters in turkeys. J. Vet. Med. A Physiol. Pathol. Clin. Med.

[36] Sunu P, Sunarti D, Mahfudz L.D, Yunianto V.D (2021). Effect of synbiotic from *Allium sativum* and *Lactobacillus acidophilus* on hematological indices, antioxidative status and intestinal ecology of broiler chicken. J. Saudi Soc. Agric. Sci..

[37] Tóthová C, Sesztáková E, Bielik B, Nagy O (2019). Changes of total protein and protein fractions in broiler chickens during the fattening period. Vet. World.

[38] Awad W.A, Ghareeb K, Abdel-Raheem S, Böhm J (2009). Effects of dietary inclusion of probiotic and synbiotic on growth performance, organ weights, and intestinal histomorphology of broiler chickens. Poult. Sci.

[39] Mohiti-Asli M, Ghanaatparast-Rashti M (2018). Comparing the effects of a combined phytogenic feed additive with an individual essential oil of oregano on intestinal morphology and microflora in broilers. J. Appl. Anim. Res.

[40] Chacher M, Kamran Z, Ahsan U, Ahmad S, Koutoulis K, Ud Din H.Q, Cengiz Ö (2017). Use of mannan oligosaccharide in broiler diets:An overview of underlying mechanisms. Worlds Poult. Sci. J.

